# Mutations within *FGFR1* are associated with superior outcome in a series of 83 diffuse midline gliomas with *H3F3A* K27M mutations

**DOI:** 10.1007/s00401-020-02259-y

**Published:** 2021-01-12

**Authors:** Ulrich Schüller, Peter Iglauer, Mario M. Dorostkar, Christian Mawrin, Jochen Herms, Armin Giese, Markus Glatzel, Julia E. Neumann

**Affiliations:** 1 Institute of Neuropathology, University Medical Center Hamburg-Eppendorf, Hamburg, Germany; 2 Department of Pediatric Hematology and Oncology, University Medical Center Hamburg-Eppendorf, Hamburg, Germany; 3 Research Institute Children’s Cancer Center Hamburg, Hamburg, Germany; 4 Institute of Pathology, University Medical Center Hamburg-Eppendorf, Hamburg, Germany; 5 Center for Neuropathology, Ludwig-Maximilians-University, Munich, Germany; 6 German Center for Neurodegenerative Diseases, Munich, Germany; 7 Institute of Neuropathology, University Hospital Magdeburg, Magdeburg, Germany

Diffuse midline glioma (DMG), H3 K27M mutant (WHO grade IV) is listed as a separate CNS tumor entity since 2016 [5], after large sequencing efforts had discovered H3 K27M mutations frequently appearing in gliomas located in midline structures [11]. Over time, we and others have observed single cases of DMG with concomitant mutations within *FGFR1* or *BRAF* [1, 2, 4, 6, 7, 9, 10, 12–14]. *FGFR1* and *BRAF* mutations are typical hallmarks of low grade glioma, such as pilocytic astrocytoma, ganglioglioma, or dysembryoplastic neuroepithelial tumor [3, 8]. So, the parallel occurrence of H3 and *FGFR1/BRAF* mutations within a single tumor may complicate the diagnostic decision towards a low grade or a high grade glioma. This dilemma, which has direct clinical implications, is particularly evident, if only small biopsies are taken and low-grade histology may not be respresentative and hence may not mirror the biology of the neoplasm. On the other hand, the presence of a MAPK pathway alteration, such as *FGFR1* or *BRAF* mutations, may open up additional possibilities of targeted therapies, independent of the tumor classification.

In order to learn more about the frequency and impact on such mutations, we analyzed a series of 83 DMG, *H3F3A* K27M mutant. Details on clinical characteristics of patients are listed in Fig. 1a and Supplementary Table 1, online resource. One case (1.2%) displayed a *BRAF* (p.V600E) mutation and 9/83 cases (10.8%) showed *FGFR1* mutations *(*p.K656E or p.N546K). Mutations within *NF1, TP53,* and *ATRX* were detected in 31.8%, 51.4%, and 35.2%, respectively. *TP53* mutations were significantly associated with *FGFR1* wild type status (*FGFR1* WT, *p* = 0.009, *Χ*^2^-test, Supplementary Fig. 1a, online resource).

**Fig. 1:**
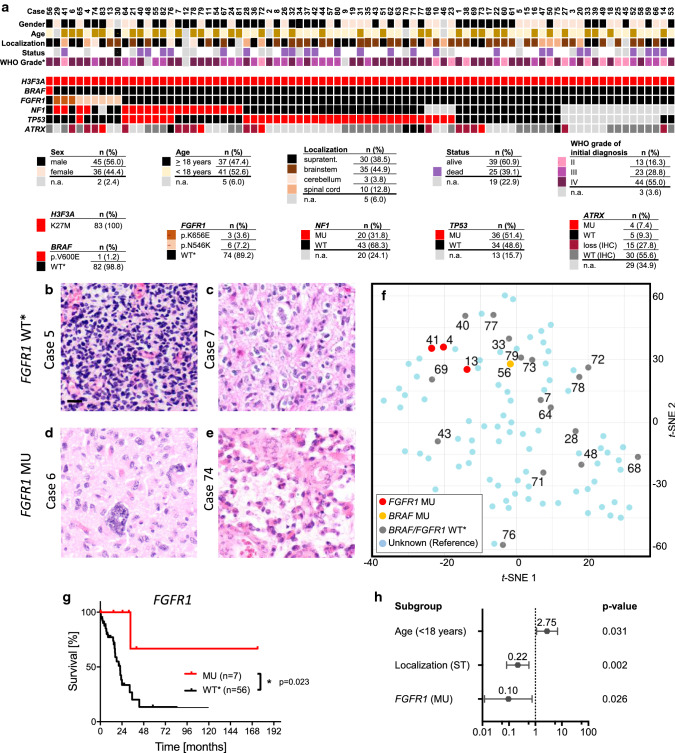
Clinical, histological, and molecular parameters of *H3F3A* K27M mutated DMG with and without additional mutations in *FGFR1*. **a** Overview on all 83 analyzed cases with 12% of cases harboring *BRAF* or *FGFR1* hotspot mutations. Percentages of characteristics refer to cases with known attribute only. Representative images of *FGFR1* WT (**b**, **c)** and MU cases (**d**, **e)** demonstrate comparable histomorphology in both groups. *T*-SNE analysis of DMG reveals *FGFR1* and *BRAF* MU cases to harbor similar DNA methylation profiles as *FGFR1* and *BRAF* WT cases (**f**). *FGFR1* MU cases showed a significantly better prognosis than *FGFR1* WT cases (*p* = 0.023, g), and multivariate analyses confirmed significance of *FGFR1* status independent of age and localization. WT* = wild type for respective hotspot, MU = mutant, n. a. = not available, *WHO grade of initial diagnosis

Similar to *FGFR1* WT cases, cases with additional *FGFR1* mutation displayed features of a diffusely growing glioma with increased cellularity and signs of anaplasia, such as increased cell pleomorphism, mitoses, or vessel proliferation (Fig. 1b-e). Furthermore, all analyzed *FGFR1* MU cases (and the *BRAF* MU case) matched to the methylation class”DMG, H3 K27M mutant” (Supplementary Fig. 1b, online resource, Fig. 1f, Supplementary Table 1, online resource).

Higher age (≥ 18 years), supratentorial tumor localization and *FGFR1* MU status were associated with a significantly better prognosis of patients (*p* = 0.038, *p* = 0.034, and *p* = 0.023, Fig. 1g and Supplementary Fig. 2a, b, online resource). In contrast, *TP53* MU status was associated with a significantly worse prognosis of patients (*p* = 0.002, Supplementary Fig. 2c, online resource). Including the latter factors in a multivariate cox regression analyses showed localization and *TP53* status as significant variables (Supplementary Fig. 2d, online resource). *FGFR1* and *TP53* mutations occurred almost mutually exclusive and hence did not represent independent variables (see also Supplementary Fig. 1a, online resource). Thus, we performed a multivariate analysis including the independent variables age, localization, and *FGFR1* status only (Fig. 1h). In this context, *FGFR1* MU status was significantly associated with a better overall survival, independently of patient age, and tumor localization (*p* = 0.026). Interestingly, the single patient (#56) with an accompanying BRAF p.V600E mutation remained alive at 24.5 months after initial diagnosis. However, the prognosis for such diffuse midline gliomas with dual H3F3A p.K27M and BRAF p.V600E mutations remains to be defined.

Together, our results suggest that RAS-MAPK-pathway signaling might play an important role in DMG with implications for diagnosis, prognosis, and therapy of respective patients.

## Supplementary Information

Below is the link to the Supplementary Information.

Supplementary file 1 (PPTX 3817 kb)

## Data Availability

Global DNA Methylation data have been deposited under GEO accession number GSE161944.
