# Supplementary material for: Mutations within FGFR1 are associated with superior outcome in a series of 83 diffuse midline gliomas with H3F3A K27M mutations
Source: Acta Neuropathol. 2021 Jan 12;141(2):323–5. doi: 10.1007/s00401-020-02259-y (PMC7847449; doi:10.1007/s00401-020-02259-y)
Supplement: Supplementary file 1 — (PPTX 3817 kb) [file 401_2020_2259_MOESM1_ESM.pptx]

## Slide 1
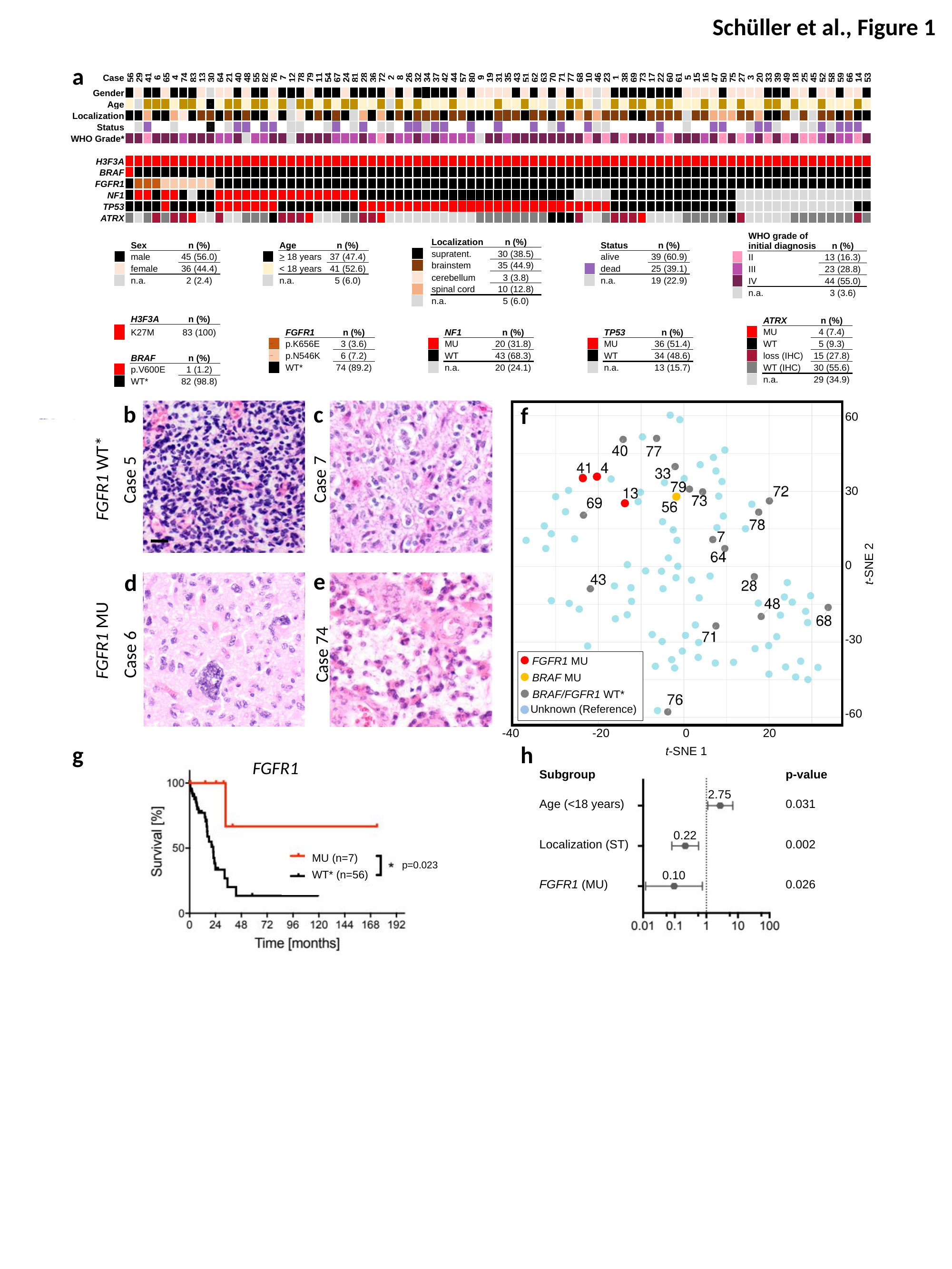

Schüller et al., Figure 1
a
| Case | 56 | 29 | 41 | 6 | 65 | 4 | 74 | 83 | 13 | 30 | 64 | 21 | 40 | 48 | 55 | 82 | 76 | 7 | 12 | 78 | 79 | 11 | 54 | 67 | 24 | 81 | 28 | 36 | 72 | 2 | 8 | 26 | 32 | 34 | 37 | 42 | 44 | 57 | 80 | 9 | 19 | 31 | 35 | 43 | 51 | 62 | 63 | 70 | 71 | 77 | 68 | 10 | 46 | 23 | 1 | 38 | 69 | 73 | 17 | 22 | 60 | 61 | 5 | 15 | 16 | 47 | 50 | 75 | 27 | 3 | 20 | 33 | 39 | 49 | 18 | 25 | 45 | 52 | 58 | 59 | 66 | 14 | 53 |
| --- | --- | --- | --- | --- | --- | --- | --- | --- | --- | --- | --- | --- | --- | --- | --- | --- | --- | --- | --- | --- | --- | --- | --- | --- | --- | --- | --- | --- | --- | --- | --- | --- | --- | --- | --- | --- | --- | --- | --- | --- | --- | --- | --- | --- | --- | --- | --- | --- | --- | --- | --- | --- | --- | --- | --- | --- | --- | --- | --- | --- | --- | --- | --- | --- | --- | --- | --- | --- | --- | --- | --- | --- | --- | --- | --- | --- | --- | --- | --- | --- | --- | --- | --- |
| Gender | m | f | m | m | f | m | m | m | f | NA | f | f | m | f | m | m | f | m | m | m | f | m | m | m | f | m | m | m | m | f | m | f | m | m | m | m | m | f | m | f | f | f | f | m | f | m | f | m | f | m | f | f | NA | f | m | m | m | m | m | m | m | m | f | f | f | f | m | f | f | f | f | m | m | m | f | f | m | f | f | m | f | f | m |
| Age | adolescent | NA | adult | adult | adult | adolescent | adult | adult | adolescent | adult | adolescent | adult | adult | adolescent | adult | adult | adolescent | adult | NA | adult | adult | adolescent | adult | adolescent | adult | adult | adolescent | adolescent | adult | NA | adult | adolescent | adult | adolescent | adolescent | adolescent | adult | adolescent | adolescent | adolescent | adolescent | adult | adolescent | adolescent | adult | adolescent | adolescent | NA | adolescent | adult | adult | adolescent | NA | adolescent | adult | adolescent | adult | adult | adolescent | adult | adult | adolescent | adolescent | adolescent | adult | adolescent | adult | adolescent | adult | adolescent | adolescent | adult | adult | adolescent | adult | adolescent | adolescent | adult | adolescent | adolescent | adolescent | adult | adolescent |
| Localization | ST | ST | SP | ST | ST | SP | CB | ST | BS | BS | ST | BS | ST | BS | ST | ST | CB | ST | NA | CB | ST | BS | ST | BS | ST | NA | SP | ST | SP | ST | BS | ST | BS | BS | BS | ST | BS | BS | ST | ST | ST | BS | BS | BS | ST | BS | BS | ST | BS | ST | SP | BS | SP | BS | BS | BS | ST | ST | BS | BS | BS | BS | NA | BS | BS | SP | SP | SP | BS | BS | SP | ST | ST | BS | NA | BS | NA | BS | BS | ST | BS | ST | ST |
| Status | 0 | NA | 1 | 0 | 0 | NA | 0 | 0 | 0 | 0 | 0 | NA | 1 | 1 | 0 | 1 | 1 | 0 | NA | NA | 0 | 0 | NA | 1 | 0 | NA | 1 | 0 | NA | NA | 0 | 1 | 1 | NA | 1 | 1 | 0 | 0 | 1 | 0 | 0 | 1 | 0 | 0 | 0 | 1 | 0 | NA | 1 | 0 | 0 | 1 | NA | NA | 0 | 0 | 0 | 0 | 0 | 1 | 0 | 0 | NA | 0 | 0 | 1 | 1 | 0 | 0 | NA | 1 | 1 | NA | 0 | 0 | NA | NA | 1 | NA | 1 | 1 | 1 | 0 |
| WHO Grade\* | IV | IV | II | IV | IV | IV | III | IV | IV | IV | III | III | IV | NA | III | III | IV | IV | NA | IV | IV | IV | IV | III | III | III | IV | III | II | IV | III | III | IV | III | IV | III | III | III | III | NA | IV | IV | III | IV | IV | IV | IV | IV | IV | IV | IV | II | IV | II | IV | II | IV | IV | IV | III | II | IV | IV | IV | III | IV | II | IV | II | III | IV | II | IV | II | IV | II | II | III | IV | III | III | II | IV |
| | | | | | | | | | | | | | | | | | | | | | | | | | | | | | | | | | | | | | | | | | | | | | | | | | | | | | | | | | | | | | | | | | | | | | | | | | | | | | | | | | | | |
| H3F3A | MU | MU | MU | MU | MU | MU | MU | MU | MU | MU | MU | MU | MU | MU | MU | MU | MU | MU | MU | MU | MU | MU | MU | MU | MU | MU | MU | MU | MU | MU | MU | MU | MU | MU | MU | MU | MU | MU | MU | MU | MU | MU | MU | MU | MU | MU | MU | MU | MU | MU | MU | MU | MU | MU | MU | MU | MU | MU | MU | MU | MU | MU | MU | MU | MU | MU | MU | MU | MU | MU | MU | MU | MU | MU | MU | MU | MU | MU | MU | MU | MU | MU | MU |
| BRAF | MU | WT | WT | WT | WT | WT | WT | WT | WT | WT | WT | WT | WT | WT | WT | WT | WT | WT | WT | WT | WT | WT | WT | WT | WT | WT | WT | WT | WT | WT | WT | WT | WT | WT | WT | WT | WT | WT | WT | WT | WT | WT | WT | WT | WT | WT | WT | WT | WT | WT | WT | WT | WT | WT | WT | WT | WT | WT | WT | WT | WT | WT | WT | WT | WT | WT | WT | WT | WT | WT | WT | WT | WT | WT | WT | WT | WT | WT | WT | WT | WT | WT | WT |
| FGFR1 | WT | p.K656E | p.K656E | p.K656E | p.N546K | p.N546K | p.N546K | p.N546K | p.N546K | p.N546K | WT | WT | WT | WT | WT | WT | WT | WT | WT | WT | WT | WT | WT | WT | WT | WT | WT | WT | WT | WT | WT | WT | WT | WT | WT | WT | WT | WT | WT | WT | WT | WT | WT | WT | WT | WT | WT | WT | WT | WT | WT | WT | WT | WT | WT | WT | WT | WT | WT | WT | WT | WT | WT | WT | WT | WT | WT | WT | WT | WT | WT | WT | WT | WT | WT | WT | WT | WT | WT | WT | WT | WT | WT |
| NF1 | WT | MU | MU | WT | MU | MU | WT | NA | WT | WT | MU | MU | MU | MU | MU | MU | MU | MU | MU | MU | MU | MU | MU | MU | MU | MU | WT | WT | WT | WT | WT | WT | WT | WT | WT | WT | WT | WT | WT | WT | WT | WT | WT | WT | WT | WT | WT | WT | WT | WT | NA | NA | NA | NA | WT | WT | WT | WT | WT | WT | WT | WT | WT | WT | WT | WT | WT | WT | NA | NA | NA | NA | NA | NA | NA | NA | NA | NA | NA | NA | NA | NA | NA |
| TP53 | WT | WT | WT | WT | MU | WT | WT | WT | WT | WT | MU | MU | MU | MU | MU | MU | MU | WT | WT | WT | WT | WT | WT | WT | WT | WT | MU | MU | MU | MU | MU | MU | MU | MU | MU | MU | MU | MU | MU | MU | MU | MU | MU | MU | MU | MU | MU | MU | MU | MU | MU | MU | MU | MU | WT | WT | WT | WT | WT | WT | WT | WT | WT | WT | WT | WT | WT | WT | NA | NA | NA | NA | NA | NA | NA | NA | NA | NA | NA | NA | NA | WT | WT |
| ATRX | WT | NA | WT | loss | WT | loss | loss | MU | NA | NA | loss | NA | NA | WT | WT | WT | WT2 | loss | loss | loss | MU | NA | NA | NA | WT | WT | loss | loss | MU | NA | NA | NA | NA | NA | NA | NA | NA | NA | NA | WT | WT | WT | WT | WT | WT | WT | WT | WT2 | WT2 | WT2 | loss | NA | NA | WT | loss | loss | loss | MU | NA | NA | NA | NA | WT | WT | WT | WT | WT | WT2 | loss | NA | NA | NA | NA | NA | WT | WT | WT | WT | WT | WT | WT | loss | WT |
| | | Localization | n (%) |
| --- | --- | --- | --- |
| WT | | supratent. | 30 (38.5) |
| BS | | brainstem | 35 (44.9) |
| CB | | cerebellum | 3 (3.8) |
| SP | | spinal cord | 10 (12.8) |
| NA | | n.a. | 5 (6.0) |
| | | WHO grade of initial diagnosis | n (%) |
| --- | --- | --- | --- |
| II | | II | 13 (16.3) |
| III | | III | 23 (28.8) |
| IV | | IV | 44 (55.0) |
| NA | | n.a. | 3 (3.6) |
| | | Sex | n (%) |
| --- | --- | --- | --- |
| WT | | male | 45 (56.0) |
| f | | female | 36 (44.4) |
| NA | | n.a. | 2 (2.4) |
| | | Age | n (%) |
| --- | --- | --- | --- |
| WT | | > 18 years | 37 (47.4) |
| adolescent | | < 18 years | 41 (52.6) |
| NA | | n.a. | 5 (6.0) |
| | | Status | n (%) |
| --- | --- | --- | --- |
| 0 | | alive | 39 (60.9) |
| 1 | | dead | 25 (39.1) |
| NA | | n.a. | 19 (22.9) |
| | | H3F3A | n (%) |
| --- | --- | --- | --- |
| MU | | K27M | 83 (100) |
| | | ATRX | n (%) |
| --- | --- | --- | --- |
| MU | | MU | 4 (7.4) |
| WT | | WT | 5 (9.3) |
| loss | | loss (IHC) | 15 (27.8) |
| WT | | WT (IHC) | 30 (55.6) |
| NA | | n.a. | 29 (34.9) |
| | | FGFR1 | n (%) |
| --- | --- | --- | --- |
| p.K656E | | p.K656E | 3 (3.6) |
| p.N546K | | p.N546K | 6 (7.2) |
| WT | | WT\* | 74 (89.2) |
| | | NF1 | n (%) |
| --- | --- | --- | --- |
| MU | | MU | 20 (31.8) |
| WT | | WT | 43 (68.3) |
| NA | | n.a. | 20 (24.1) |
| | | TP53 | n (%) |
| --- | --- | --- | --- |
| MU | | MU | 36 (51.4) |
| WT | | WT | 34 (48.6) |
| NA | | n.a. | 13 (15.7) |
| | | BRAF | n (%) |
| --- | --- | --- | --- |
| MU | | p.V600E | 1 (1.2) |
| WT | | WT\* | 82 (98.8) |
b
c
FGFR1 WT*
Case 7
Case 5
e
d
FGFR1 MU
f
60
30
t-SNE 2
0
-30
FGFR1 MU
BRAF MU
BRAF/FGFR1 WT*
Unknown (Reference)
-60
-40
-20
0
20
t-SNE 1
Case 6
Case 74
g
h
FGFR1
| Subgroup | | p-value |
| --- | --- | --- |
| Age (<18 years) | | 0.031 |
| Localization (ST) | | 0.002 |
| FGFR1 (MU) | | 0.026 |
2.75
0.22
p=0.023
MU (n=7)
WT* (n=56)
0.10
